# Beamforming Techniques for Passive Radar: An Overview

**DOI:** 10.3390/s23073435

**Published:** 2023-03-24

**Authors:** José M. Núñez-Ortuño, José P. González-Coma, Rubén Nocelo López, Francisco Troncoso-Pastoriza, María Álvarez-Hernández

**Affiliations:** Defense University Center at the Spanish Naval Academy, 36920 Marín, Spain

**Keywords:** passive radar, beamforming, cross ambiguity function, uniform linear array, MRC, MVDR, ZF

## Abstract

Passive radar is an interesting approach in the context of non-cooperative target detection. Because the signal source takes advantage of the so-called illuminator of opportunity (IoO), the deployed system is silent, allowing the operator cheap, portable, and practically undetectable deployments. These systems match perfectly with the use of antenna arrays to take advantage of the additional gains provided by the coherent combination of the signals received at each element. To obtain these benefits, linear processing methods are required to enhance the system’s performance. In this work, we summarize the main beamforming methods in the literature to provide a clear picture of the current state of the art. Next, we perform an analysis of the benefits and drawbacks and explore the chance of increasing the number of antenna elements. Finally, we identify the major challenges to be addressed by researchers in the future.

## 1. Introduction

In recent years, the use of passive radar that utilizes existing signals in the environment as illuminators of opportunity (IoOs) has gained attention in the academic and military fields due to its capability to detect targets without revealing the location of the sensor, because it does not emit radar signals [1]. Therefore, its receiver is much less susceptible to electronic countermeasures (ECM) [2], and its intrinsic bistatic operation has anti-stealth capabilities. Furthermore, as it is completely passive, deploying the receiver does not need frequency allocation, making it usable in densely populated areas where electromagnetic interference can be a problem, nor does it require a careful transmit beamforming design to avoid interception [3].

Passive radars are also smaller and less expensive than their active counterparts. Examples of signals that can be used as IoOs in passive radar include frequency modulation (FM) radio [4,5], digital audio broadcasting (DAB) [6] and digital video broadcasting–terrestrial (DVB-T) [7,8], global system for mobile communications (GSM) [9,10] and long term evolution (LTE) [11], global navigation satellite system (GNSS) [12,13,14] and digital video broadcasting–satellite (DVB-S) [15], WLAN [16], and 5G [17,18,19] signals. Because the waveforms transmitted by these opportunity transmitters are not optimized for radar applications and there is a lack of control over the transmission parameters, complex signal processing methods are required to detect the weak scattering signal from the targets.

### 1.1. Passive Bistatic Radar Scenario

**Figure 1:**
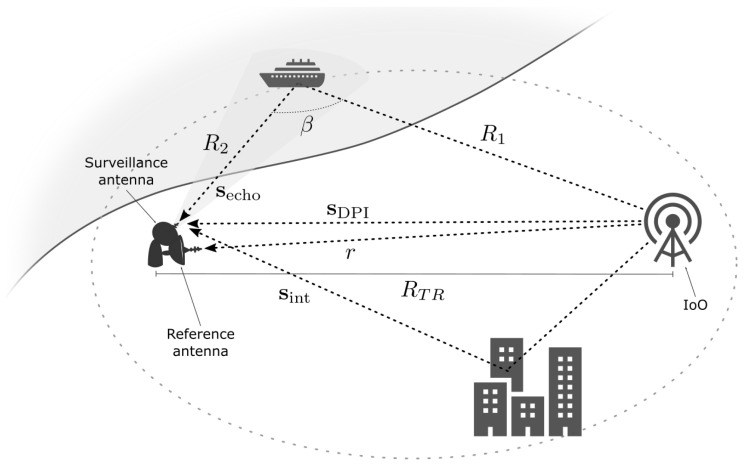
Basic bistatic passive radar scenario.

Figure 1 depicts the basic scheme of a bistatic passive radar, where $R_{1}$ is the target-to-transmitter distance, $R_{2}$ is the target-to-receiver distance, $R_{TR}$ is the transmitter-to-receiver distance or baseline, which is known in advance, and β is the bistatic angle defined by $R_{1}$ and $R_{2}$.

The different techniques available for target detection rely on a comparison between a direct signal coming from the IoO and the echo signals reflected by the targets. In the context of passive radar, this assumption results in a receiver that is equipped with two different channels, namely the reference channel for capturing the reference signal, r(t), and the surveillance channel for capturing the surveillance signal, s(t). The received surveillance signal comprises the direct component from the IoO, $s_{\mathrm{DPI}}\left(t\right)$, the echo reflected from the target, $s_{\mathrm{echo}}\left(t\right)$, and additional echo terms corresponding to potential clutter, multipath, and/or extra targets, $s_{\mathrm{int}}\left(t\right)$.

The system performance is significantly affected by the direct-path interference (DPI) signal, $s_{\mathrm{DPI}}\left(t\right)$, captured by the surveillance channel arriving from the IoO. This interference correlates perfectly with the reference signal and has a level that can be significantly higher than the level of the echoes from the targets.

Unlike classical radar approaches, where the transmitter and receiver are located in the same position, the passive radar technique estimates the so-called bistatic range, $R=R_{1}+R_{2}-R_{TR}$. Likewise, passive radar can be used to calculate the bistatic velocity of targets. Note that bistatic range and velocity values are not directly comparable to their monostatic equivalents. The use of the bistatic range instead of the monostatic range is due to the uncertainty introduced by the geometrical model inherent to this kind of system. It is well known that a bistatic range measurement places the target on an ellipsoid (Figure 1), with the transmitter and receiver located at its foci (iso-range contour).

Target positions are obtained by measuring the direction of arrival (DoA) of the target echo signal in addition to the range measurement. The target is located at the intersection of the ellipsoid and the estimated DoA cone. Another option for locating a target is to use multiple transmitter–receiver pairs to calculate the point of intersection of the ellipsoids, using one transmitter and multiple receivers or multiple transmitters and one receiver [20].

In the first solution, the DoA can be estimated using an array of antennas at the receiver for the surveillance channel. If the elements are spaced appropriately, a directive pattern can be generated through the collective processing of signals received at each element. This DoA estimation approach has the advantage of improving the signal-to-noise ratio (SNR) by combining energy from multiple antennas (beamforming) and allows the beam to be electronically directed in any direction, or even a set of directional beams to be generated that comprehensively cover the entire area of interest (AoI) in the air space. This would necessarily require the use of multiple coherent reception channels.

Several solutions have been proposed to obtain DoA information, e.g., beam scanning [21] or super-resolution methods [22,23]. Nevertheless, we assume that the angular direction of the target is known, as these estimation methods fall out of the scope of the present work.

Beamforming techniques can also be used to collect the transmitted signal by synthesizing a single beam pointed at an IoO or multiple beams pointed to multiple disparate IoOs, or to take advantage of this space–time characterization enabling clutter/multipath cancellation filters.

As noted above, this opportunistic illuminator might broadcast any type of broadband signal, for instance, FM radio, DVB-T, DAB, GSM, LTE, etc. All of them are common candidates for providing the opportunistic waveforms required for passive radar systems. The choice of one type of signal (waveform) of opportunity is based on many aspects, including the type of target and its dynamics, the expected range and velocity resolution, the system coverage, and obviously, the availability of the IoOs.

In bistatic passive radar applications, the minimum range separation required between two targets is called range resolution ΔR, where two targets are assumed to be co-linear with the bistatic bisector. The range resolution is defined as follows:

(1) $$\Delta R=\frac{c}{2B\cos(\beta /2)},$$

where *B* and *c* are the signal bandwidth and the light velocity, respectively, and β is the bistatic angle defined in Figure 1.

For a fixed bistatic angle, the bistatic range resolution is determined by the IoO signal bandwidth. Range resolution is inversely proportional to the baseband bandwidth of the transmitted waveform. Assuming that $\beta =60^{\circ }$, typical resolutions of 1.0–3.5 km may be achieved with FM radio signals, 800 m with GSM, 100 m with DAB, 22 m with DVB-T, 9 m with LTE, and 7 m with GNSS.

The Doppler resolution in passive radar can be determined from the receiver’s period of time that signal is acquired, termed coherent processing interval (CPI) and denoted by $T_{\mathrm{CPI}}$. The CPI duration determines how the radar can best observe targets of different radial velocities. The bistatic velocity resolution can be defined as

(2) $$\Delta v=\frac{\lambda }{2T_{\mathrm{CPI}}\cos\left(\beta /2\right)},$$

where λ is the carrier wavelength.

Assuming again a fixed bistatic angle and a specific value of $T_{\mathrm{CPI}}$, the best velocity resolutions are obtained for the highest carrier frequencies. Typical values of velocity resolution obtained with $\beta =60^{\circ }$ and $T_{\mathrm{CPI}}=0.25$ s are 7 m/s with FM radio signals, 3 m/s with DAB, 0.8 m/s with DVB-T, 0.5 with GNSS, 0.4 m/s with GSM, 0.25 m/s with LTE, and 0.07 m/s with DVB-S.

The range or coverage of the system depends on the transmit power of the IoO. For a given target and bistatic geometry, the higher the transmit power, the larger the bistatic range targets can be detected. FM and DVB-T are the most powerful emitters and GNSS and DVB-S are the weakest.

### 1.2. Prior Art

Cross ambiguity function is one key indicator that is commonly used to evaluate radar performance for a specific signal or waveform. In passive radar, target detection and parameter estimation are traditionally performed by evaluating the cross ambiguity function (CAF), i.e., correlating s(t) with delayed and Doppler shifted versions of the reference transmit signal r(t). In bistatic passive radar, CAF shape can be determined by two factors: system geometry (position and direction of the target motion) and waveform properties.

Multiple solutions have been proposed in passive radar to enhance target detection and identify its DoA using adaptive beamforming techniques in order to improve localization performances. In this paper, we will evaluate the different beamforming options and their applicability to a variety of practical passive radar scenarios. These solutions can be carried out in the angular domain if the spatial processing is performed prior to the CAF or in the range–Doppler domain if the CAF is performed before the adaptive beamforming. In the angular domain, well-known solutions are available in the radar literature [24], which can be employed depending on the figure of merit to be optimized. These solutions will be termed maximum ratio combining (MRC) [25], minimum variance distortionless response (MVDR) [26], and zero-forcing (ZF) [25,27], and we will present their main advantages and drawbacks, as well as an analysis regarding their utilization in the context of passive radar. These techniques require accurate knowledge of the angular locations to offer good performance. Unfortunately, this knowledge is difficult to acquire. When the signal is processed in the range–Doppler domain, the estimation of the covariance matrices to use spatial filtering techniques is computed using scenario information regarding the range and Doppler features of the interference. Other options leverage temporal processing to remove interference, such as the extensive cancellation algorithm (ECA) [28] and other solutions, but they are severely affected when multipath components are present or rely on particular waveforms to operate [29], which is impractical in the context of passive radars.

In the angular domain, the array response vector calculation method is derived in its most general form from the spatial covariance matrix of the data received during the CPI [29]. However, this procedure presents two potential drawbacks. In the first place, the DoA of the target needs to be accurately known, otherwise, it could be canceled unintentionally. Second, in order to calculate correctly the beamforming vector, the spatial covariance matrix should only contain interference signal components. However, in the majority of practical cases, it is not possible to separate the interference component from the signal component. Thenceforth, countless adaptive beamforming techniques have been proposed in order to improve the spatial covariance matrix estimation. In [30], the reconstruction of the interference-plus-noise covariance matrix using the spatial spectrum distribution and the correction of the array response vector was proposed to maximize the beamformer output power without converging to any interference. In [31,32], the decomposition of the spatial covariance matrix using eigen-subspace-based beamforming techniques to the signal and noise subspaces was proposed, where the influence of the surveillance signal can be eliminated in the covariance matrix using a side-lobe canceller. In [27], the estimate of the spatial covariance matrix combining deterministic sidelobe reduction and adaptive pattern shaping that preserves the low sidelobe level is discussed. An intermediate solution between the angular domain and the range–Doppler domain can be found in [33], where adaptive beamforming is applied after the range–Doppler processing has been processed, whereas the sample matrix inversion method has been previously used in the angular domain to estimate the noise plus interference spatial covariance matrix.

Additional solutions to improve the adaptive beamforming techniques based on the fully dimensional space–time domain, known as space–time adaptive processing (STAP) techniques, have been studied in [34]. These schemes combine the information corresponding to different dimensions to characterize and mitigate interference. These techniques are referred to as STAP and explore a particular angle–range–Doppler combination at a time [35,36]. Once this combination is selected, an artificial vector is computed by stacking several angular responses for the chosen range–Doppler bin together with a set of range responses for fixed angle–Doppler values. The performance of this kind of approach is, however, heavily dependent on the particular scenario, as the authors assume a spread-Doppler clutter and interference in the neighborhood of the target and the absence of the target for the same range–Doppler region, or the utilization of a particular waveform in the IoO [34]. Moreover, the construction of the aforementioned vectors, as well as their size, is conducted based on experiments [35] and thus is complicated to analyze in a general context. For these reasons, we will concentrate on the general versions based on spatial filtering whether selecting a Doppler or a range region. Regarding the beamforming selection, in this kind of work, the method to optimize the signal-to-interference-plus-noise ratio (SINR) is using a minimum variance space–time adaptive beamforming followed by a least squares spatial adaptive filtering.

In the range–Doppler domain, the first approach of adaptive beamforming techniques was proposed for high-frequency over-the-horizon passive radar [29]. This method tries to avoid the target echo cancellation problem inherent to the angular domain processing. An alternative solution based on spatial adaptive beamforming after the cross-correlation function was proposed in [37,38]. In [37], a processing method containing spatial filtering and high resolution DoA estimators working in the range–Doppler domain was proposed. In [38], a spatial smoothing beamforming-based Bucci algorithm was proposed to suppress the direct reference signal and the clutter, while the well-known multiple signal classification (MUSIC) algorithm [22] was used to estimate the angle of arrival of the target return by searching the array response that is orthogonal to a noise subspace, which is obtained from singular value decomposition of the range–Doppler matrix. Practical implementations of adaptive beamforming techniques in the range–Doppler domain can be found in [39,40]. Therein, the authors employed difference covariance matrices based on range or Doppler characterizations of the targets and compare with a strategy referred to as deterministic null, where a ZF beamformer is pointed to the directions where interference sources produce strong radar returns.

The aim of this work is to describe the main beamforming approaches in the context of passive radar, focusing on their particular features regarding usability, side information required for its utilization, the expected performance results, and computational costs. Moreover, we will explore how these solutions apply to the different domains of the received signal and compare the benefits and drawbacks of both solutions. In addition, as an actual trend in the context of radar and communications is an increase in the number of elements on the array to radiate narrow energy beams, we provide some insight concerning the effects of moving to large dimensional arrays in a passive radar setup. Finally, we identify some limitations of the proposed methods and explore future lines of research that might be of interest to the scientific community.

After this introductory part (Section 1), the paper is structured as follows: the system model and the main beamforming approaches are presented in Section 2. Section 3 develops the above techniques applied in different domains (beamforming before and after the CAF). Section 4 contains the results of simulations showing the behavior and gains of the different techniques and a subsequent discussion of them. Finally, open questions and lines of future research are explained in Section 5, and Section 6 contains the main conclusions of this work.

Notation: Lower and upper case bold letters denote vectors and matrices, and $C^{M}$ is complex vector space with dimension *M*; ${\left(\cdot \right)}^{T}$, ${\left(\cdot \right)}^{H}$ denote the transpose and Hermitian transpose operations, respectively; ℜ{·} and ℑ{·} represent the real and the imaginary parts of a complex number; symbol∼reads as *statistically distributed as* and $E\left[\cdot \right]$ is the statistical expectation; $N_{C}\left(0,C\right)$ is a zero-mean Gaussian distribution with covariance C; ∥·∥ is the Euclidean norm.

## 2. System Model

In this work, we focus on the scenario where the IoO is equipped with a single antenna. In the point of interest, the passive radar system is located to receive the aforementioned signal. However, we separately deploy a single antenna for capturing the reference signal r(t) and an antenna array of *M* elements to acquire the surveillance signal $s\left(t\right)\in C^{M}$.

**Figure 2:**
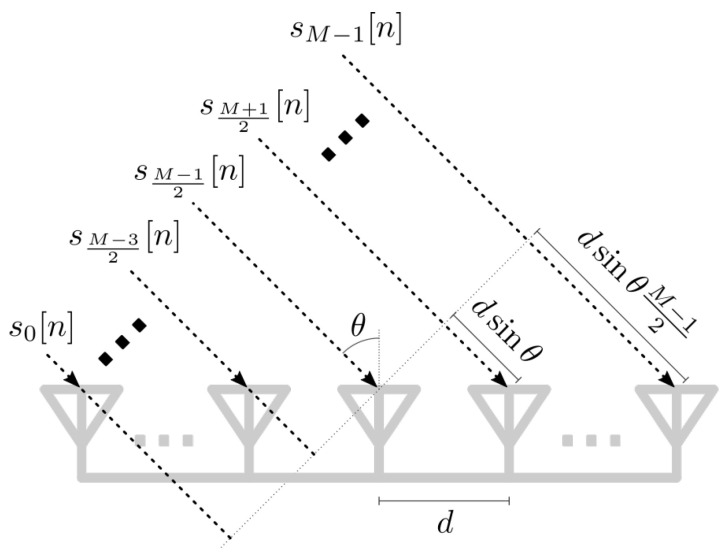
Structure of an ULA arrangement with *M* antennas.

In particular, we consider that the baseband complex data signal is given by $x\left(t\right)e^{j2\pi f_{c}t}\in C$, with carrier frequency $f_{c}$ and signal bandwidth *B*. This signal passes through the reference and surveillance channels, for which we only consider the line-of-sight (LoS) component, and is acquired at the sampling frequency $f_{s}=\frac{1}{T_{s}}$. Thus, for the reference channel, we produce the discrete time samples at instant $t=nT_{s}$ described as follows:

(3) r[n]=αx[n]+w[n],

with w[n]∈C being the additive white Gaussian noise (AWGN) and α∈C the complex gain collecting several propagation effects. We assume that the reference signal is isolated and, therefore, there are no multipath components. During the CPI, $N_{\mathrm{CPI}}=T_{\mathrm{CPI}}f_{s}$ samples are captured. Simultaneously, we acquire the signal corresponding to the surveillance channel, which contains the direct signal and the echo reflected from a moving target, as follows:

(4) $$\begin{array}{cc} s[n] & =s_{\mathrm{DPI}}\left[n\right]+s_{\mathrm{echo}}\left[n\right]+s_{\mathrm{int}}\left[n\right]+z\left[n\right]\\ \; & =\underset{\mathrm{DPI}}{\underbrace{\alpha_{d}a\left(\theta_{d}\right)x\left[n-\tau_{d}\right]}}+\underset{\mathrm{desired}\;\mathrm{signal}}{\underbrace{\alpha_{e}a\left(\theta_{e}\right)x\left[n-\tau_{e}\right]e^{j2\pi \frac{f_{D}}{f_{s}}n}}}+\underset{\mathrm{interference}\;\mathrm{signals}}{\underbrace{\sum_{i=0}^{N_{i}-1}\alpha_{i}a\left(\theta_{i}\right)x\left[n-\tau_{i}\right]e^{j2\pi \frac{f_{D_{i}}}{f_{s}}n}}}+z\left[n\right], \end{array}$$

where $\alpha_{d},\alpha_{e},\alpha_{i}\in C$, are the complex attenuation introduced by the channel, $\tau_{d}$, $\tau_{e}$, and $\tau_{i}$ are time delays, expressed in samples, and we have introduced the Doppler frequency shift $f_{D}=\frac{v}{\lambda }$, with $\lambda =\frac{c}{f_{c}}$. Moreover, as the surveillance signal is obtained by means of an antenna array, the spatial distribution of the elements has to be taken into consideration. Hence, we denote the array response vector by $a\left(\theta \right)\in C^{M}$ for the angular direction θ, and introduce $\theta_{d}$, $\theta_{e}$, and $\theta_{i}$ to represent the angular directions for the direct, echo, and interference paths, respectively. In addition, we denote the noise vector as $z\left[n\right]\;\;∼\;\;N_{C}\left(0,\sigma^{2}I_{M}\right)$. The additional $N_{i}$ echo terms in (4) correspond to multi-path components, clutter, and/or further targets that may be present in the scenario, with their associated attenuation coefficients, angular directions, delays, and Doppler shifts. Finally, by assuming an uniform linear array (ULA) arrangement, as presented in Figure 2, a(θ) reads as [41]

(5) $$a\left(\theta \right)={\left[e^{-j\frac{2\pi }{c}f_{c}d\sin\left(\theta \right)\frac{M-1}{2}},e^{-j\frac{2\pi }{c}f_{c}d\sin\left(\theta \right)\left(\frac{M-1}{2}-1\right)},\ldots ,e^{j\frac{2\pi }{c}f_{c}d\sin\left(\theta \right)\frac{M-1}{2}}\right]}^{T},$$

where *d* is the inter-element distance and *M* is odd for notation simplicity. The use of different array geometries, mainly rectangular and circular, is possible in the context of passive radar, and results in array response vectors a(θ) showing its own particular spatial features [42].

It is important to highlight that the surveillance signal in (4) is a *M*-element vector, where the entries exhibit a strong correlation due to the spatial closeness of the antenna elements. As such, the received signals can be linearly combined using the beamforming vector b, that is,

(6) $$y\left[n\right]=b^{H}s\left[n\right].$$

This feature enables the usage of different beamforming techniques depending on the desired system features.

### 2.1. System Performance Metrics

An important aspect of any system, as it will provide a figure of merit to establish comparisons, is the performance metric chosen to evaluate a design. In this work, to measure the kindness of the proposed solutions, we will employ two well-known performance metrics, viz., SNR and SINR. These metrics consider a desired signal d(t), a noise component n(t), and *K* more interference sources $i_{k}\left(t\right)$, k∈{1,…,K}. The available signal is then $a\left(t\right)=d\left(t\right)+\sum_{k=1}^{K}i_{k}\left(t\right)+n\left(t\right)$, and the performance metrics read as

(7) $$\begin{array}{cc} \mathrm{SNR} & =\frac{E[|d\left(t\right){|}^{2}]}{E[|n\left(t\right){|}^{2}]}, \end{array}$$

(8) $$\begin{array}{cc} \mathrm{SIN}R & =\frac{E[|d\left(t\right){|}^{2}]}{{E[|n\left(t\right)|}^{2}]+\sum_{k=1}^{K}E[|i_{k}\left(t\right){|}^{2}]}. \end{array}$$

While the SNR focuses on the ratio between the powers of the signal of interest and the noise, the SINR also takes into account the effects of potential interference signals that might disrupt the proper reception of the received signal. Usually, the beamforming designs are oriented to optimize one of the later metrics, or a variation of any of them.

### 2.2. Main Beamforming Strategies

In this section, we analyze the main beamforming approaches used in radar systems. These techniques provide designs based on different criteria that can be employed depending on the particular features of the AoI. For example, they can be used to enhance the strength of the signal of interest or to remove interference or other undesired effects, among others. These promising characteristics are achieved by jointly combining the received signals with a linear transformation with expression (6) and assuming that b is constant during a CPI interval. Therefore, in this work we will distinguish among three major approaches for the design of the beamforming vector b, namely, MRC, MVDR [24], and ZF [25]. The details on the optimization problem that they solve and their respective expressions are presented in Table 1, where we have introduced the desired direction d, interference matrix R, and the normalization factor ρ. The particular values of d and R are given by the practical setup and the domain where the spatial dimension of the received signal is employed, as discussed in the ensuing sections. Next, we provide some insight regarding these different options for beamforming.

#### 2.2.1. MRC

MRC is a basic strategy that consists of maximizing the power of the desired signal d. In addition to its simplicity, this approach presents other benefits with respect to more elaborated options. First, the calculation of the beamformer is very efficient in terms of computational complexity and, second, the only information required to obtain the beamformer is the spatial signature of the desired signal. The main counterpart of this approach is that, as no other spatial directions are considered in the design (i.e., the influence of R in the system performance is neglected), this approach might also increase the interference when the projection of d in the subspace generated by the columns of R is non-negligible.

Although it is clear that MRC is a naïve strategy, it constitutes a very interesting option in scenarios where the desired signal and the interference are separable, as all the spatial degrees of freedom are dedicated to increasing the SNR.

#### 2.2.2. MVDR

The second design considered, MVDR, is more sophisticated as it not only enhances the desired signal strength but also mitigates the effects of undesired interference [26]. This is due to the nature of the optimization problem maximized when using this approximation, that is,

(9) $$\max\frac{|b^{H}{d|}^{2}}{b^{H}Rb}.$$

When we develop the numerator in (9) as $b^{H}dd^{H}b$, the formulation is identical to a generalized Rayleigh quotient, which is a well-known problem in the wireless communications literature [43]. This formulation aims at maximizing the ratio between the desired signal and the undesired interference or noise. For the scenario considered, $dd^{H}$ is a rank 1 Hermitian matrix, and R has to be a positive definite matrix. Accordingly, the solution to this problem is obtained as the generalized eigenvector of R and $dd^{H}$, that is, $R^{-1}dd^{H}b=\lambda_{\max}b$. Since $dd^{H}$ is a rank 1 matrix that can be decomposed as a vector product, $d^{H}b$ is a scalar, and the previous expression clearly leads to the solution $b=R^{-1}d$. The usage of this beamformer is meaningful if we intend to remove the spatial characteristics of the interference while trying to enhance the signal of interest. This is in contrast with the MRC scheme that neglects the interference matrix R.

As a counterpart, to effectively use this method, it is mandatory to acquire an accurate knowledge of the interference matrix R and the desired direction d. In addition, the computational complexity of this method scales with $O(M^{3})$, which might be impractical depending on the number of antenna elements in the array and/or the computation power of the receiver device.

#### 2.2.3. ZF

ZF is a popular option in the wireless communication literature, as it is able to completely remove the interference [44]. Thus, in multi-user scenarios where the inter-user interference is the system bottleneck, the use of ZF leads to asymptotically optimal solutions [45]. This nice feature can be also exploited in the context of passive radar to remove the DPI, multipath effects, or even clutter [27]. To achieve interference-removing capabilities, the optimization problem of ZF imposes the constraint $b^{H}R=0$, with $R\in C^{M\times K}$ being a rectangular matrix such that the number of interference sources *K* fulfills K<M. Therefore, the ZF solution is readily obtained as the orthogonal projection of the desired direction d into the null space spanned by the columns of R. Note that, although both MVDR and ZF take into account the interference, it is handled in different ways. Whereas the former strategy “corrects” the direction vector d to avoid the influence of the interference, ZF totally removes the vector components of d lying in the interference subspace. Accordingly, situations with tolerable levels of interference are not handled properly using ZF, which unnecessarily sacrifices spatial degrees of freedom to totally remove these effects. Thus, ZF appears as a good option in scenarios with strong interference and d lying in a subspace partially or not overlapping with that spanned by R.

Similar to MVDR, the benefits of employing ZF only apply when accurate knowledge of the interference matrix R and the direction d is available. Again, the computational complexity of this method is generally dominated by a matrix inversion. However, the inverse operates over a square matrix of dimension *K*, with K<M, and computational cost in the order $O(K^{3})$. If the number of antennas *M* is considerably larger than *K*, the use of ZF would be computationally more efficient than MVDR, providing a more practical approach in certain scenarios.

## 3. Beamforming Techniques to Enhance the Surveillance Signal

**Figure 3:**
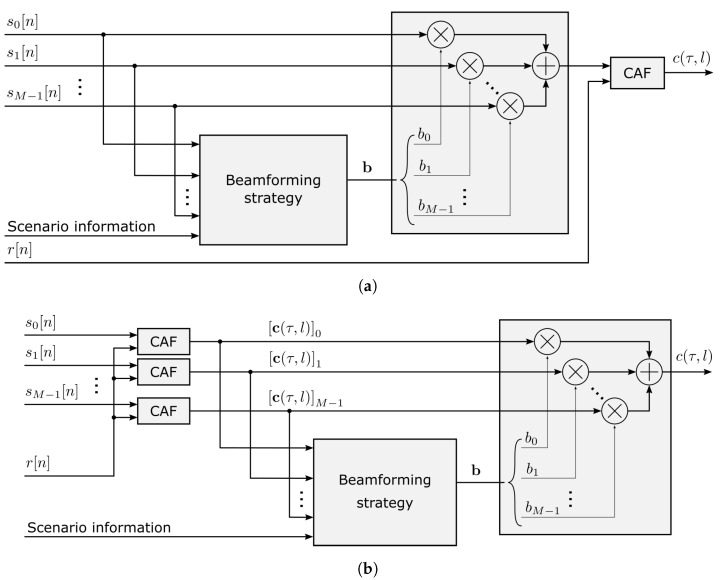
Comparison of main beamforming techniques, applied in different domains: (**a**) in the angular domain, before the CAF; (**b**) in the range–Doppler domain, after the CAF.

In the previous section, we revised different options for linearly combining the signals corresponding to the antenna elements of the array. In the context of passive radar, the combination can be performed not only in the spatial domain, where the signal and interference locations are characterized by an angular direction [29,30], but also in the range–Doppler domain, where the objects can be classified according to their distance, angle, and speed, subsequently applying beamforming techniques to improve the system performance [29,39]. The schematic views corresponding to both approaches can be seen in Figure 3. In the following, we will explore these two alternatives and analyze the opportunities arising when the information of all the antenna elements is jointly processed. Finally, we will analyze the benefits and drawbacks of these strategies.

### 3.1. Beamforming before the CAF

As already noted, an essential step in a passive radar system is to perform the comparison of the surveillance signal s[n] in (4) with the scalar signal r[n] in (3) to discover the distance and speed information related to the potential targets. In this first approach, before performing the aforementioned comparison, we use some signal processing techniques that coherently combine the signals received by the antenna array. This way, for each sample *n*, the receiver performs a linear combination and generates an effective scalar signal (6), with the aim of amplifying the target signal and/or removing undesired effects. In addition, these desirable features improve by adding more antenna elements, as the number of observations also increases. This straightforward option naturally arises in the context of passive radar, as it presents several benefits, such as SNR enhancement, interference cancellation, clutter mitigation, or DPI removal. Moreover, these kinds of advantages are commonly exploited in communications systems, where the usage of multiple antennas is one of the technologies enabling future wireless communication systems [24,46].

Let us introduce the received signal of (6) in the context of passive radar. Recall that the echo delay $\tau_{e}$ and Doppler frequency shift $f_{D}$ in (4) are unknown and have to be determined. To that end, a search is performed over a grid of bistatic range, τ, and Doppler, *l*, bins. This grid has to be defined over the delay (in samples) $T=[\tau_{\min},\tau_{\max}]$ and Doppler shift (integer) L=[−L,L] intervals, for a positive integer $L\in Z_{+}$; these intervals are chosen according to the scenario, such that $\tau_{e}\in T$, and $f_{D}\in f_{s}\left[\frac{-L}{N_{\mathrm{CPI}}},\frac{L}{N_{\mathrm{CPI}}}\right]$. At this point, it is important to remark that the bistatic range and velocity resolutions of the map depend on the system parameters according to Equations (1) and (2), respectively. Under these conditions, the relationship between the reference and the surveillance signal can be exploited. Indeed, the surveillance signal is a time-delayed and Doppler-shifted version of the reference signal. As a consequence, the techniques intended to detect and characterize non-cooperative targets take advantage of the correlation between the signals on Equations (3) and (4). Therefore, finding local maxima in the CAF is the centerpiece of passive radar signal processing [47].

After coherently combining the signals received by the antenna array, we evaluate the CAF over y[n] in (6) and the reference signal r[n] in (3), i.e.,

(10)c(τ,l)=∑n=0NCPI−1y[n]r*[n−τ]e−j2πlNCPIn(11)=α*αdbHa(θd)∑n=0NCPI−1x[n−τd]x*[n−τ]e−j2πlNCPIn(12)+α*αebHa(θe)∑n=0NCPI−1x[n−τe]x*[n−τ]e−j2πlNCPI−fDfsn(13)+α*∑n=0NCPI−1bHz[n]x*[n−τ]e−j2πlNCPIn+∑n=0NCPI−1y[n]w*[n−τ]e−j2πlNCPIn.

As already introduced, the choice of the beamformer vector b determines the amount of interference through the factor $b^{H}a\left(\theta_{d}\right)$ and the SNR of the surveillance signal with $b^{H}z\left[n\right]$. Similarly, it might help to alleviate or even remove the effects of the clutter components or additional interferers, when they are present. These extra components would lead to additional terms in the form of (11) with their respective angular directions θ. Finally, the last term in (13) contains the noise contribution and exhibits that the SNR of the reference signal is independent of the choice of b, as expected. In conclusion, the design of the beamformer vector b is key to producing a convenient range–velocity map that will later be employed to estimate the values for the delay ${\hat{\tau }}_{e}$ and Doppler frequency ${\hat{f}}_{D}$, i.e.,

(14) $$\left\{{\hat{\tau }}_{e},{\hat{f}}_{D}\right\}=\underset{\tau \in T,l\in L}{\mathrm{argmax}}\;\left|c\left(\tau ,l\right)\right|.$$

We see from (10) that |c(τ,l)| present peaks for $\tau \approx \tau_{e}$ and $l\approx \frac{f_{D}}{f_{s}}N_{\mathrm{CPI}}$ associated with the desired signal, and $\tau \approx \tau_{d}$ and l=0 corresponding to the DPI. Clearly, SNR and SINR provide a hint regarding how much the peaks of interest stand out.

Considering the received signal (6), we now evaluate the different beamforming options in Section 2.2 and their applicability to different practical scenarios. To perform this comparison, we work under the assumption that prior information regarding the target and the interference sources is available. As already stated, such information has to be estimated in practical setups, and a lot of solutions have been proposed to obtain DoA information, e.g., beam scanning [21] or super-resolution methods [22,23]. Nevertheless, we assume that the angular directions for the target and the interference signals are known, as these estimation methods fall out of the scope of the present work.

For the MRC beamformer, we assume that $\theta_{e}$ is available at the receiver. Then, the coherent processing of the received signals might exploit this knowledge by employing the beamforming vector

(15) $$b=\frac{1}{\sqrt{M}}a\left(\theta_{e}\right).$$

Then, the post-combined signal y[n] is given as follows:

(16) $$\begin{array}{c} y\left[n\right]=\alpha_{e}\sqrt{M}x\left[n-\tau_{e}\right]e^{j2\pi \frac{f_{D}}{f_{s}}n}+\alpha_{d}x\left[n\right]b^{H}a\left(\theta_{d}\right)+\frac{1}{\sqrt{M}}\sum_{m=0}^{M-1}e^{j\frac{2\pi }{c}f_{c}d\sin\left(\theta_{e}\right)\left(m-\frac{M-1}{2}\right)}z_{m}\left[n\right]. \end{array}$$

As introduced in Section 2.2, the MRC strategy points the beamformer vector to the target angular direction aiming at improving the SNR, which is defined in (7) as the quotient of the desired signal power and the noise power, that is,

(17) $${\mathrm{SNR}}_{\mathrm{MRC}}=\frac{E[|\alpha_{e}b^{H}a\left(\theta_{e}\right)x\left[n-\tau_{e}\right]e^{j2\pi \frac{f_{D}}{f_{s}}n}{|}^{2}]}{E[|b^{H}{z\left[n\right]|}^{2}]}=\frac{M|\alpha_{e}{|}^{2}E[{\left|x\left[n\right]\right|}^{2}]}{\sigma^{2}}=\frac{M|\alpha_{e}{|}^{2}}{\sigma^{2}}$$

under the common assumption $E[{\left|x\left[n\right]\right|}^{2}]=1$. In particular, the SNR increases linearly with the number of elements in the antenna array, *M*. Thus, a simple beamforming strategy is appealing due to the capacity of enhancing the CAF term associated with the target echo (12), whereas spatially white noise in (13) does not obtain any gain. Unfortunately, it is possible that the coherent combination of the signals received at each element also increases undesired interference terms, such as (11), that can be much more powerful that the ones corresponding to potential targets. Thus, the SINR metric (8), which is an extension of the SNR that also includes the interference, might constitute a more appealing figure of merit for the AoI, that is,

(18) $$\begin{array}{cc} {\mathrm{SIN}R}_{\mathrm{MRC}} & =\frac{E[|\alpha_{e}b^{H}a\left(\theta_{e}\right)x\left[n-\tau_{e}\right]e^{j2\pi \frac{f_{D}}{f_{s}}n}{|}^{2}]}{E[|b^{H}{z\left[n\right]|}^{2}]+E[|\alpha_{d}b^{H}a\left(\theta_{d}\right)x\left[n\right]{|}^{2}]}\\ \; & =\frac{|\alpha_{e}{|}^{2}E[{\left|x\left[n\right]\right|}^{2}]{\left|b^{H}a\left(\theta_{e}\right)\right|}^{2}}{b^{H}(\sigma^{2}I_{M}+{\left|\alpha_{d}\right|}^{2}E[{\left|x\left[n\right]\right|}^{2}]a\left(\theta_{d}\right)a^{H}\left(\theta_{d}\right))b}=\frac{M|\alpha_{e}{|}^{2}}{\sigma^{2}+\frac{|\alpha_{d}{|}^{2}}{M}{\left|a{\left(\theta_{e}\right)}^{H}a\left(\theta_{d}\right)\right|}^{2}}, \end{array}$$

where we have substituted the beamformer expression for the MRC to show that the interference power increases linearly with the number of antennas *M* as well.

Contrary to MRC, when the beamforming design is performed according to the MVDR, the spatial degrees of freedom available at the receiver are also used to combat interference. In such a case, not only the knowledge about the target angular direction $\theta_{e}$ is necessary, but also the angular information regarding the interfering signals. This DoA awareness might be obtained in a training stage if the interference source locations are fixed, for instance, the DPI contribution angular direction $\theta_{d}$. As ill-conditioned matrices R are unfeasible for the MVDR problem formulation of (9), a regularization term is often employed to correct rank deficient issues. Accordingly, a good choice is to define this matrix as in the next expression:

(19) $$R=|\alpha_{d}{|}^{2}a\left(\theta_{d}\right)a^{H}\left(\theta_{d}\right)+\sigma^{2}I_{M},$$

where more terms can be included for additional interfering sources with their corresponding gains and angular directions. Using this definition, the MVDR beamformer turns out to be

(20) $$b=\rho {\left(|\alpha_{d}{|}^{2}a\left(\theta_{d}\right)a^{H}\left(\theta_{d}\right)+\sigma^{2}I_{M}\right)}^{-1}a\left(\theta_{e}\right),$$

with ρ being a normalization factor ensuring ${∥b∥}^{2}=1$. The former beamformer is optimal in terms of the SINR, as we see in the next equation:

(21) $$\begin{array}{cc} {\mathrm{SIN}R}_{\mathrm{MVDR}} & =\frac{|\alpha_{e}{|}^{2}E[{\left|x\left[n\right]\right|}^{2}]{\left|b^{H}a\left(\theta_{e}\right)\right|}^{2}}{b^{H}(\sigma^{2}I_{M}+{\left|\alpha_{d}\right|}^{2}E[{\left|x\left[n\right]\right|}^{2}]a\left(\theta_{d}\right)a^{H}\left(\theta_{d}\right))b}={\left|\alpha_{e}\right|}^{2}a^{H}\left(\theta_{e}\right)R^{-1}a\left(\theta_{e}\right), \end{array}$$

where we have used b in (20) to obtain the last equality. Remarkably, when $|\alpha_{d}{|}^{2}\ll \sigma^{2}$, R results in a scaled identity matrix, and the MVDR beamformer is equivalent to the MRC one.

Another alternative to taking into consideration the detrimental effects of interference is the ZF beamformer. Similar to MVDR, the knowledge of the angular directions of the signal of interest and the interference signals is mandatory to effectively use this approach. However, the interference is handled in a different way in the two schemes. First, let us introduce the matrix R for the ZF design as

(22) $$R=[a\left(\theta_{1}\right),a\left(\theta_{2}\right),\ldots ,a\left(\theta_{K}\right)].$$

This way ZF removes the interference of K<M sources and $R\in C^{M\times K}$. In the particular case of (4), where only DPI is considered, this matrix reduces to the M×1 vector $R=a(\theta_{d})$. Therefore, the ZF beamformer is then

(23) $$b=\rho P^{⊥}a\left(\theta_{e}\right)=\rho \left(I_{M}-\frac{1}{M}a\left(\theta_{d}\right)a^{H}\left(\theta_{d}\right)\right)a\left(\theta_{e}\right),$$

that is, the orthogonal projection of the MRC beamformer into the null space of R, up to some scaling ρ. We have introduced the orthogonal projection matrix $P^{⊥}$ for notation simplicity. By plugging in Equation (23) in the SINR expression we get

(24) $$\begin{array}{cc} {\mathrm{SIN}R}_{\mathrm{ZF}} & =\frac{E[|\alpha_{e}b^{H}a\left(\theta_{e}\right)x\left[n-\tau_{e}\right]e^{j2\pi \frac{f_{D}}{f_{s}}n}{|}^{2}]}{E[|b^{H}{z\left[n\right]|}^{2}]+E[|\alpha_{d}b^{H}a\left(\theta_{d}\right)x\left[n\right]{|}^{2}]}=\frac{|\alpha_{e}{|}^{2}}{\sigma^{2}}a^{H}\left(\theta_{e}\right)P^{⊥}a\left(\theta_{e}\right). \end{array}$$

Note that, as the interference is removed thanks to the use of the ZF beamformer, the last expression shows an SNR gain as in (17). As a consequence, the cost of removing the interference by utilizing this approach is a smaller SNR gain compared to MRC, i.e., $a^{H}\left(\theta_{e}\right)P^{⊥}a\left(\theta_{e}\right)\leq M$.

#### Asymptotic Regime

In this section, we analyze the effects of one current trend in both radar and communication research areas, that is, the benefits related to the increase in the number of elements in the array. The interest of the research community goes even in the direction of mixing these two applications, as radar localization capabilities are very useful to assist channel training stages of multiple-input multiple-output (MIMO) wireless communication systems [48,49,50]. These two approaches are commonly coupled with the use of antenna arrays with a large number of elements *M*. Therefore, it is interesting to analyze the effect of incorporating this technology into the beamforming designs of passive radar systems.

Let us start by evaluating the consequences of increasing the number of elements *M* in the context of MRC beamforming. To that end, we introduce the function that computes the inner products of the array response vectors $\mu \left(\vartheta_{1},\vartheta_{2}\right)=a{\left(\vartheta_{1}\right)}^{H}a\left(\vartheta_{2}\right)$. An insightful expression for this product is given by

(25) $$\begin{array}{c} \mu \left(\vartheta_{1},\vartheta_{2}\right)=\frac{\sin\left(M\pi \frac{d}{\lambda }\left(\sin\vartheta_{2}-\sin\vartheta_{1}\right)\right)}{\sin\left(\pi \frac{d}{\lambda }\left(\sin\vartheta_{2}-\sin\vartheta_{1}\right)\right)}. \end{array}$$

Using (25), we rewrite the signal after MRC beamforming, (16), as

(26) $$\begin{array}{c} y\left[n\right]=\alpha_{e}\sqrt{M}x\left[n-\tau_{e}\right]e^{j2\pi \frac{f_{D}}{f_{s}}n}+\alpha_{d}x\left[n\right]\frac{\mu (\theta_{e},\theta_{d})}{\sqrt{M}}+\frac{1}{\sqrt{M}}\sum_{m=0}^{M-1}e^{j\frac{2\pi }{c}f_{c}d\sin\left(\theta_{e}\right)\left(m-\frac{M-1}{2}\right)}z_{m}\left[n\right]. \end{array}$$

Particularly, in the asymptotic regime where M→∞, the second term in (26) cancels out except when $\theta_{d}=\theta_{e}$, as the numerator of (25) is bounded, whereas the denominator sublinearly increases with *M*. Moreover, notice that the noise vector contains independent Gaussian distributed variables $z_{m}\left[n\right]$, m∈{0,…,M−1}, with a variance decreasing on *M*. Thus, the third term of (26) also reduces with *M*, and the received signal approaches to $y\left[n\right]\approx \alpha_{e}\sqrt{M}x\left[n-\tau_{e}\right]e^{j2\pi \frac{f_{D}}{f_{s}}n}$, which is a scaled version of the desired signal in (4). Likewise, the SINR reduces to

(27) $$\begin{array}{cc} {\mathrm{SIN}R}_{\mathrm{MRC}} & =\frac{M|\alpha_{e}{|}^{2}}{\sigma^{2}+\frac{|\alpha_{d}{|}^{2}}{M}{\left|a{\left(\theta_{e}\right)}^{H}a\left(\theta_{d}\right)\right|}^{2}}=\frac{M|\alpha_{e}{|}^{2}}{\sigma^{2}+\frac{|\alpha_{d}{|}^{2}}{M}\mu {\left(\theta_{e},\theta_{d}\right)}^{2}}\approx \frac{M|\alpha_{e}{|}^{2}}{\sigma^{2}}, \end{array}$$

which is the SNR expression in (17). As such, MRC is the optimal choice in the asymptotic regime.

From our previous discussion, it is clear that when the number of available antenna elements is small, the clutter, multi-path, and DPI cancellation features of the asymptotic regime do not apply unless more sophisticated beamforming designs such as MVDR or ZF are employed. However, as the available degrees of freedom reduce in this scenario, the interference removal capabilities spoil the beamforming gain for the desired signal. Now, we show that the three solutions converge when we increase the number of antenna elements, i.e., MVDR and ZF beamformers approach the MRC one. In the case of MVDR this can be noticed by expanding the matrix inverse in (20), yielding

(28) $$\begin{array}{cc} b & =\rho {\left(|\alpha_{d}{|}^{2}a\left(\theta_{d}\right)a^{H}\left(\theta_{d}\right)+\sigma^{2}I_{M}\right)}^{-1}a\left(\theta_{e}\right)\\ \; & =\rho \left[\frac{1}{\sigma^{2}}I_{M}-\frac{|\alpha_{d}{|}^{2}}{\sigma^{4}}a\left(\theta_{d}\right){\left(1+\frac{|\alpha_{d}{|}^{2}}{\sigma^{2}}a^{H}\left(\theta_{d}\right)a\left(\theta_{d}\right)\right)}^{-1}a^{H}\left(\theta_{d}\right)\right]a\left(\theta_{e}\right)\\ \; & =\frac{\rho }{\sigma^{2}}a\left(\theta_{e}\right)-\rho \frac{|\alpha_{d}{|}^{2}}{\sigma^{4}}a\left(\theta_{d}\right){\left(1+\frac{|\alpha_{d}{|}^{2}}{\sigma^{2}}M\right)}^{-1}\mu \left(\theta_{e},\theta_{d}\right)\approx \frac{\rho }{\sigma^{2}}a\left(\theta_{e}\right), \end{array}$$

where we have used the matrix inversion lemma [51]. By properly adjusting the scaling factor ρ, we obtain (15). Following the same lines, the performance metric ${\mathrm{SIN}R}_{\mathrm{MVDR}}$ reduces to

$$\begin{array}{cc} {\mathrm{SIN}R}_{\mathrm{MVDR}} & =|\alpha_{e}{|}^{2}a^{H}\left(\theta_{e}\right)R^{-1}a\left(\theta_{e}\right)\\ \; & =\frac{|\alpha_{e}{|}^{2}}{\sigma^{2}}a^{H}\left(\theta_{e}\right)a\left(\theta_{e}\right)-\frac{|\alpha_{d}{|}^{2}}{\sigma^{4}}{\left(1+\frac{|\alpha_{d}{|}^{2}}{\sigma^{2}}M\right)}^{-1}\mu^{2}\left(\theta_{e},\theta_{d}\right)\approx \frac{M|\alpha_{e}{|}^{2}}{\sigma^{2}}, \end{array}$$

which is the SNR expression for MRC beamforming in (17).

In the case of ZF, we rewrite (23) to again evaluate the impact of asymptotically large values of *M*; this is

$$b=\rho P^{⊥}a\left(\theta_{e}\right)=\rho a\left(\theta_{e}\right)-\rho \frac{1}{M}\mu \left(\theta_{e},\theta_{d}\right)a\left(\theta_{d}\right)\approx \rho a\left(\theta_{e}\right).$$

Similar to MVDR, in the case of ZF, we obtain the MRC beamformer when we increase the number of antenna elements. For the performance metric ${\mathrm{SIN}R}_{\mathrm{ZF}}$ in (24), we get

$$\begin{array}{c} {\mathrm{SIN}R}_{\mathrm{ZF}}=\frac{|\alpha_{e}{|}^{2}}{\sigma^{2}}a^{H}\left(\theta_{e}\right)P^{⊥}a\left(\theta_{e}\right)=\frac{|\alpha_{e}{|}^{2}}{\sigma^{2}}a^{H}\left(\theta_{e}\right)a\left(\theta_{e}\right)-\frac{1}{M}\frac{|\alpha_{e}{|}^{2}}{\sigma^{2}}\mu^{2}\left(\theta_{e},\theta_{d}\right)\approx \frac{M|\alpha_{e}{|}^{2}}{\sigma^{2}}. \end{array}$$

Comparing both SINR expressions, ${\mathrm{SIN}R}_{\mathrm{MVDR}}$ and ${\mathrm{SIN}R}_{\mathrm{ZF}}$, we observe that the main difference comes from the treatment of the AWGN. Whereas ${\mathrm{SIN}R}_{\mathrm{MVDR}}$ approximates ${\mathrm{SNR}}_{\mathrm{MRC}}$ when the noise power $\sigma^{2}$ is large compared to the interference power $|\alpha_{d}{|}^{2}$, this fact is completely ignored in ${\mathrm{SIN}R}_{\mathrm{ZF}}$, which is more suited for practical situations where interference is the dominating term in the signal disturbance.

Although in this analysis we have considered the asymptotic regime, the interference cancellation capabilities of beamforming approximately hold for a large but not impractical number of antennas M≈100 [52]. From a practical point of view, observe that the antenna array size for a DVB-T signal with $d=\frac{\lambda }{2}$ under a ULA arrangement is about 25 m, and 5 m for a planar configuration. These are reasonable sizes for a building roof or a wall. When moving to higher frequencies, as in millimeter wave (mmWave) radar systems [53,54], these sizes considerably reduce, obtaining vales of about 25 cm for 60 GHz frequencies.

### 3.2. Beamforming after the CAF

An alternative approach to that described in the previous section consists of performing the CAF directly over the received signals for each element and applying the beamforming procedure afterward in the range–Doppler domain. In this scheme, the CAF is independently evaluated over each element of the array m∈[0,M−1], allowing us to work with a vector of functions c(τ,l). In opposition to the previous method, where the $\theta_{e}$ is considered to be known prior to the computation of (10), this second approach provides additional flexibility in the exploitation of the spatial features of the received signal. Taking into account the aforementioned process, the CAF vector reads as

[c(τ,l)]m=∑n=0NCPI−1s[n]mr*[n−τ]e−j2πlNCPIn=∑n=0NCPI−1αd[a(θd)]mx[n−τd]+αe[a(θe)]mx[n−τe]ej2πfDfsn+z[n]mr*[n−τ]e−j2πlNCPIn(29)=α*αdej2πcfcdsin(θd)(m−M−12)∑n=0NCPI−1x[n−τd]x*[n−τ]e−j2πlNCPIn(30)+α*αeej2πcfcdsin(θe)(m−M−12)∑n=0NCPI−1x[n−τe]x*[n−τ]e−j2π(lNCPI−fDfs)n(31)+α*∑n=0NCPI−1z[n]mx*[n−τ]e−j2πlNCPIn+∑n=0NCPI−1s[n]mw*[n−τ]e−j2πlNCPIn.

The main terms in the expression are separated in the different lines of the former equation. In particular, (29) is the term associated with the direct path, with a phase rotation that corresponds to the antenna element *m* and the angle $\theta_{d}$, (30) presents a similar structure but represents the signal corresponding target echo and, finally, (31) is composed of the error terms associated to the surveillance and reference channels, respectively.

It is clear that one of the main challenges for the schemes that aim at mitigating or removing the interference, presented in Section 3.1, is the acquisition of proper spatial interference matrices R. Among other difficulties, one must realize that, during the training steps performed to estimate these interference matrices, the signals to be rejected are usually mixed with the desired signals in the channel observations. This lack of separability might cause the rejection of target echoes with spatial characteristics similar to that of the interference signals. Furthermore, it is common that a side-effect of these cancellations leads to a reduction in the overall signal power.

When beamforming is applied after the CAF, it makes it possible to isolate the received signal contributions corresponding to the target echoes, clutter, and potential interference sources as they present different signatures in the range–Doppler domain. Therefore, the spatial characterization of unwanted signals is easier to get, as the spatial covariance matrices can be obtained from the vectors c(τ,l) associated with certain values for the range and the Doppler parameters. If a target echo presents a different range–Doppler pair than the clutter or the interference, it will be possible to build an interference matrix R that alleviates the interference without masking potential targets [29].

Recall that targets, as well as interference and clutter, usually expand over several ranges and/or Doppler bins. This fact is employed to select regions that partially represent the interference and the noise with the aim of interference mitigation, while not affecting the actual signal of interest. To that end, we can find two different approaches in the literature. The first one identifies a set of Doppler bins $L_{-}\cup L_{+}$ corresponding to interference and uses subsets corresponding to the lower $L_{-}=\left\{l_{-}^{L},l_{-}^{L}+1,\ldots ,l_{-}^{U}\right\}$ and the upper $L_{+}=\left\{l_{+}^{L},l_{+}^{L}+1,\ldots ,l_{+}^{U}\right\}$ part of this set, together with all the range cells, to define the matrices $R_{-}$ and $R_{+}$. Next, $R_{-}$ is employed to compute the beamformer with the object of sounding the upper subset of Doppler frequencies, whereas $R_{+}$ offers the change of exploring the lower subset [29]. Alternatively, all Doppler shifts might be selected for covariance matrix estimation, whereas the range would be the dimension allowing one to separate clutter and interference from the targets with the information contained in the bistatic distances. An additional advantage of this approach is that targets appear in several range bins due to movement, thus reducing the risk of unintentionally removing the desired signal masked by the interference [40].

To exhibit the spatial signature of this approach, we will use a matrix-vector notation to highlight the similarities and differences with the approach that exclusively relies on the angular domain to enhance the received signal, that is,

(32) $$\begin{array}{cc} c(\tau ,l) & \approx a\left(\theta_{d}\right)g_{d}\left(\tau ,l\right)+a\left(\theta_{e}\right)g_{e}\left(\tau ,l\right)+\dot{z}\left(\tau ,l\right), \end{array}$$

where we have used the approximation $\sum_{n=0}^{N_{\mathrm{CPI}}-1}{\left[s[n]\right]}_{m}w^{*}\left[n-\tau \right]e^{-j2\pi \frac{l}{N_{\mathrm{CPI}}}n}\approx 0$. This approach is reasonable as the reference channel is often corrected [10,55] and considered as error-free [34]. Moreover, we have introduced the scalar correlation functions $g_{d}\left(\tau ,l\right)$ and $g_{e}\left(\tau ,l\right)$, and the spatially white Gaussian random vector $\dot{z}\left(\tau ,l\right)$, defined as follows:

(33) $$\begin{array}{cc} g_{d}\left(\tau ,l\right) & =\alpha^{*}\alpha_{d}\sum_{n=0}^{N_{\mathrm{CPI}}-1}x\left[n-\tau_{d}\right]x^{*}\left[n-\tau \right]e^{-j2\pi \frac{l}{N_{\mathrm{CPI}}}n},\\ g_{e}\left(\tau ,l\right) & =\alpha^{*}\alpha_{e}\sum_{n=0}^{N_{\mathrm{CPI}}-1}x\left[n-\tau_{e}\right]x^{*}\left[n-\tau \right]e^{-j2\pi (\frac{l}{N_{\mathrm{CPI}}}-\frac{f_{D}}{f_{s}})n},\\ \dot{z}\left(\tau ,l\right) & =\alpha^{*}\sum_{n=0}^{N_{\mathrm{CPI}}-1}z\left[n\right]x^{*}\left[n-\tau \right]e^{-j2\pi \frac{l}{N_{\mathrm{CPI}}}n}. \end{array}$$

By employing Equation (32), we will analyze this scheme from a similar perspective to that of Section 3.1 to reveal the common aspects and the main differences. Let us start introducing the interference plus noise matrix associated with a particular set of Doppler bins and all the ranges

R−=∑τ∈T∑l∈L−c(τ,l)cH(τ,l)(34)=∑τ∈T∑l∈L−|gd(τ,l)|2a(θd)aH(θd)+|ge(τ,l)|2a(θe)aH(θe)(35)+z˙(τ,l)z˙H(τ,l)+2ℜ{gd*(τ,l)ge(τ,l)a(θe)aH(θd)}+2ℜ{ge*(τ,l)z˙(τ,l)aH(θe)}+2ℜ{gd*(τ,l)z˙(τ,l)aH(θd)}.

We can expect, for the structure of this matrix, a strong influence of the addends scaled by the squared absolute values of the correlation functions in (34), as well as from the noise covariance matrix obtained with the outer products of the noise vectors at the beginning of (35). On the contrary, it is expected that the remaining terms, which include crossed products, have a small effect on the covariance matrix.

If we consider a good selection of Doppler bin set $L_{-}$, we must be able to isolate the interference and noise term from the previous expression, as this choice would lead to small values for the cross-products of the scalar correlation terms for a target Doppler-spread not included in $L_{-}$, obtaining

(36) $$R_{-}\approx \sum_{\tau \in T}\sum_{l\in L_{-}}{\left|g_{d}\left(\tau ,l\right)\right|}^{2}a\left(\theta_{d}\right)a^{H}\left(\theta_{d}\right)+\dot{z}\left(\tau ,l\right){\dot{z}}^{H}\left(\tau ,l\right).$$

This matrix is then employed to build a beamforming vector that is useful to sound the upper Doppler bins, due to its capability for alleviating the effects of the interference and the noise, that is, the authors in [29] resort to the MVDR solution with

(37) $$b_{-}=\rho R_{-}^{-1}a\left(\theta_{e}\right).$$

Next, $b_{-}$ is used to produce the scalar CAF by linearly combining the *M* components of the CAF vector as $c_{+}\left(\tau ,l\right)=b_{-}^{H}c\left(\tau ,l\right)$ for $l\in L_{+}$. Analogously, $b_{+}=\rho R_{+}^{-1}a\left(\theta_{e}\right)$ is computed using $R_{+}$ for targets sought in the Doppler range $l\in L_{-}$, with $c_{-}\left(\tau ,l\right)=b_{+}^{H}c\left(\tau ,l\right)$.

Depending on the particularities of the scenario, it might be interesting to swap the roles of range and Doppler dimensions in the computation of the interference and noise matrices R. This approach was employed in [40].

We will now evaluate the SINR expression when this kind of solution is used, under the assumption of Doppler bin set $L_{-}$ properly selected

(38) $$\begin{array}{cc} {\mathrm{SIN}R}^{+}\; & =\frac{|\alpha_{e}{|}^{2}{\left|b_{-}^{H}a\left(\theta_{e}\right)\right|}^{2}}{b_{-}^{H}Rb_{-}}=\frac{|\alpha_{e}{|}^{2}{\left(a^{H}\left(\theta_{e}\right)R_{-}^{-1}a\left(\theta_{e}\right)\right)}^{2}}{a^{H}\left(\theta_{e}\right)R_{-}^{-1}RR_{-}^{-1}a\left(\theta_{e}\right)}, \end{array}$$

where R is the actual interference plus noise matrix from (19) for the set of bins $L_{+}$, and $b_{-}$ will be optimal only if $R_{-}=\gamma R$, for some scaling factor γ. Accordingly, the SINR level is bounded by that achieved with the knowledge of the interference source angles. Nevertheless, under ideal conditions, $R_{-}$ in (36) can be approximated by

(39) $$R_{-}\approx {\left|\alpha \right|}^{2}|\alpha_{d}{|}^{2}a\left(\theta_{d}\right)a^{H}\left(\theta_{d}\right)+{\left|\alpha \right|}^{2}I_{M},$$

thus achieving optimality for the SINR metric. As the nice properties of the asymptotically large *M* regime can be again derived from (36) following a similar procedure to that of the MVDR in Section 3.1, we refer to the reader to that section for further details.

### 3.3. Discussion

In the previous sections, we analyzed the characteristics of the two main approaches of beamforming for passive radar. Table 2 contains a classification of the main work in the literature with respect to these different techniques.

Regarding the comparison between performing beamforming in the angular or range–Doppler domain, we can establish the ensuing key ideas:

- Target detection and parameter estimation are more manageable in the range–Doppler domain. As already said, when the DoA estimation is performed in the angular domain, it is easier to miss the estimation of the target if its angular direction is similar to that of a strong interference source. In spite of this, obtaining the covariance matrices in the range–Doppler domain also has a downside. Indeed, the sample covariance matrices obtained with this method have to be computed with an adequate sample size to fulfill the full rank condition to avoid numerical problems and to effectively capture the spatial structure of the interference and the noise. The number of samples grows with the number of antennas *M*, which is inconvenient when moving to large antenna arrays.
- Processing in the range–Doppler domain lacks the flexibility of the angular domain approach. Apart from MVDR, it is not possible to employ other beamforming solutions that estimate the sample covariance matrices because they contain both the interference and the noise, so the ZF strategy is not applicable. This can be easily appreciated in the classification provided in Table 2.
- Regarding the computational complexity, the angular domain can take advantage of the flexibility of using different beamforming designs that might be adapted to different situations. For instance, the MRC is a useful and computationally cheap approach when interference is not strong or the antenna array is adequately orientated to mitigate it. In addition, the computational complexity of the ZF approach is dominated by a matrix inversion of the size of the number of interference sources, which might be very small compared to the number of antenna elements *M*. On the contrary, MVDR, which is the only option for beamforming in the range–Doppler domain estimating the covariance matrix, performs a matrix inversion of size *M*. When moving to larger antenna array sizes, this fact, together with the computational load introduced when the CAF is performed for each antenna element, might lead to impractical situations.
- In relation to SINR increase produced by the beamforming operation, we have clearly stated that, under favorable conditions, the SINR achieved with the range–Doppler approach is equal to that obtained with the proper angular directions. Therefore, the scenario of application and, correspondingly, the method employed to accurately obtain the angular spatial information of the setup is a key idea to choosing one approach over the other one.

## 4. Results and Discussion

This section is devoted to the numerical evaluation of the main beamforming strategies in the literature. The results obtained support the conclusions of our previous analysis and provide additional insight regarding the expected behavior of the different approaches.

### 4.1. Scenario Description

**Figure 4:**
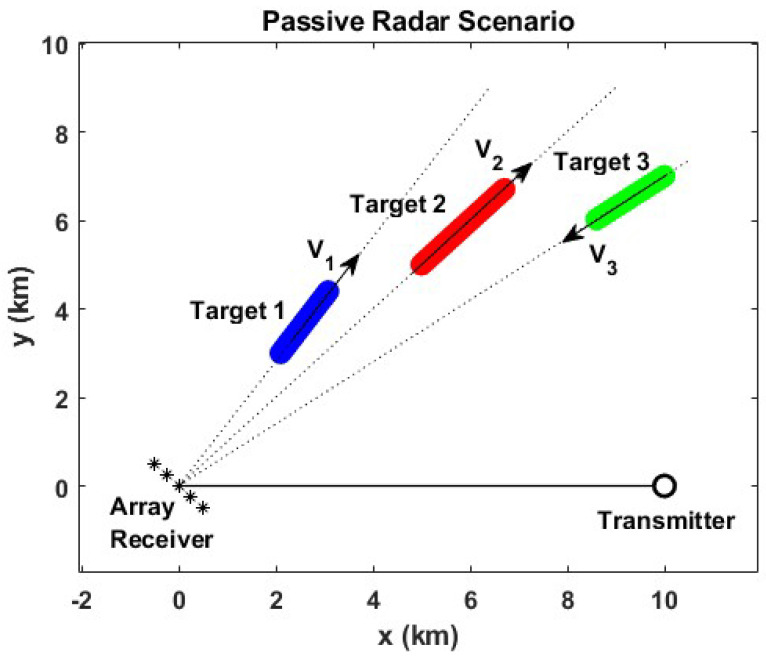
Synthetic passive radar scenario. The symbols ’*’ depict the position and the number of the array elements.

Synthetic passive radar data were generated in order to simulate a realistic scenario and evaluate the performance of the different beamforming techniques presented in the previous sections. Figure 4 shows the geometry of this scenario, whose main blocks are: the receiver antenna array, the transmitter antenna, and different targets located inside of the AoI. We consider a ULA receiver antenna composed of a variable number of individual antennas *M* that are equally spaced with a distance d=0.5λ for a frequency of 618 MHz (which corresponds to a DVB-T channel of interest for future research development). In the AoI, we introduce three targets moving at different velocities and directions ($-10^{\circ }$, $0^{\circ }$, and $10^{\circ }$ from the broadside array, respectively). Without loss of generality, the transmitter antenna is simply considered to be an omnidirectional antenna located 10 km away from the receiver antenna array.

The opportunistic waveform used in this work allows reaching a maximum range resolution ΔR of 12 m. A CPI of 85 ms was selected in order to obtain a maximum Doppler resolution of 5.7 Hz. In the next subsections, the desired signal is associated with the echo produced by target #2 of the simulated scenario, and the interference or undesired signals correspond to targets arriving from the angular locations $-10^{\circ }$ and $10^{\circ }$, associated with the echoes produced by targets #1 and #3, respectively. Furthermore, the corresponding echo powers are assumed to be of 0 dB relative to the desired signal power. The main transmission parameters used in the numerical evaluation, as well as the bistatic resolutions reached, are summarized in Table 3.

### 4.2. Evaluation of the Different Beamforming Strategies

In this section, the different beamforming approaches previously presented (MRC, MVDR, and ZF) are analyzed and compared in terms of their capabilities to enhance the signal strength in the steering angle and to remove, or at least attenuate, the interference and other undesired signals coming from other directions. Different noise power levels are considered in order to evaluate the performance of the different beamforming techniques when the spatially white noise is added to the interference in determined directions.

**Figure 5:**
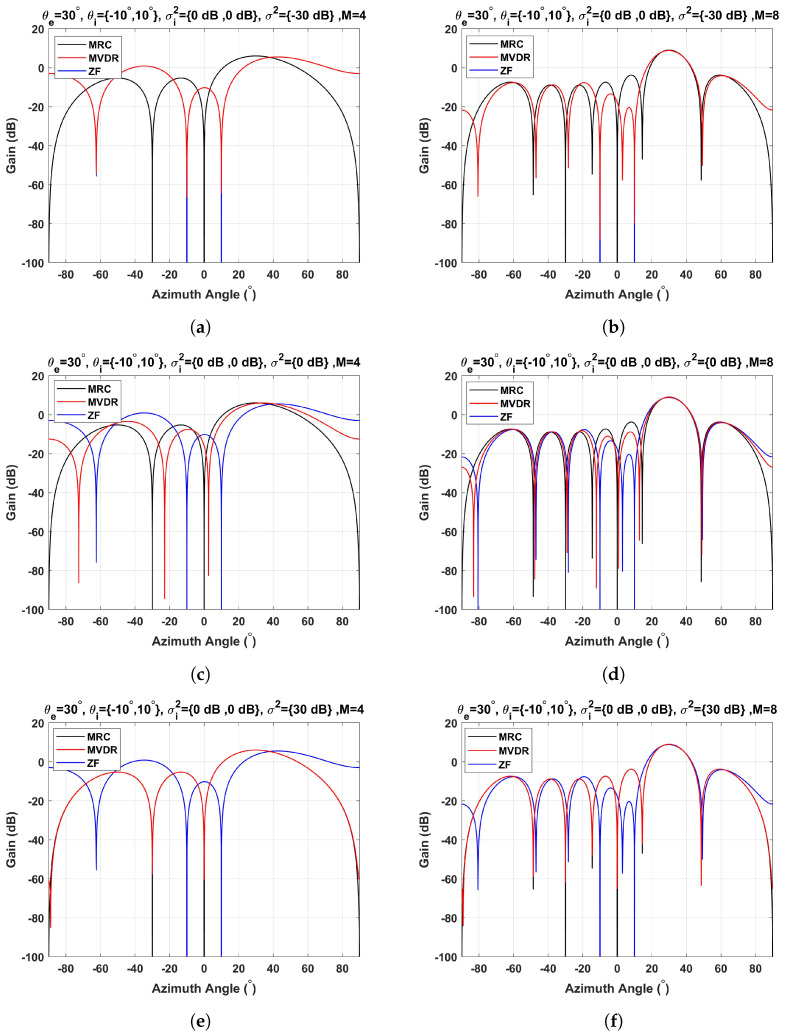
Beamforming techniques as a function of the number of array elements and the noise power (steering angle of $30^{\circ }$): (**a**) M = 4, $\sigma^{2}=-30$ dB; (**b**) M = 8, $\sigma^{2}=-30$ dB; (**c**) M = 4, $\sigma^{2}=0$ dB; (**d**) M = 8, $\sigma^{2}=0$ dB; (**e**) M = 4, $\sigma^{2}=30$ dB; (**f**) M = 8, $\sigma^{2}=30$ dB.

In Figure 5, the array response patterns for the MRC, MVDR, and ZF techniques are shown as a function of the number of array elements and the white noise power. Without loss of generality, a representative maximum steering angle of $30^{\circ }$ is considered, as it is generally limited by the individual radiant element. Two undesired target signal echoes arriving from $-10^{\circ }$ and $10^{\circ }$ with corresponding powers $\sigma_{i}^{2}$ of 0 dB relative to the desired target signal echo power are considered.

As expected, as the number of array elements increases, so does the energy concentration at the desired angular direction. Furthermore, the angular resolution is finer because the main beam is narrower, thus increasing the gain. With respect to the different techniques, both the MRC and the ZF beamformers do not depend on the noise power, hence the array response is the same for a given number of array elements. In particular, the MRC technique maximizes the power of the desired target echo signal at the steering angle, whereas the ZF technique guarantees that the power of the interfering signals in the respective arrival angles is zero. In contrast, the MVDR response is a more sophisticated technique because it maximizes the ratio between the desired signal power and the undesired signal power (interference and noise power). Figure 5 shows the MVDR response for a white noise power $\sigma^{2}$ of −30 dB, 0 dB, and 30 dB relative to the desired signal power. It should be noted that when the noise power is much smaller than the desired signal power, the MVDR response meets the ZF response (Figure 5a,b). In contrast, if the noise power is much higher than the desired signal power, the MVDR response converges to the MRC response (Figure 5e,f). However, for an intermediate situation (for example, noise power of 0 dB), MVDR maximizes the ratio between the desired signal and the combination of the interference signals and the noise. Despite this fact, the interference signals are not completely removed and the array gain is not maximum in the steering angle of the desired signal (Figure 5c,d).

**Figure 6:**
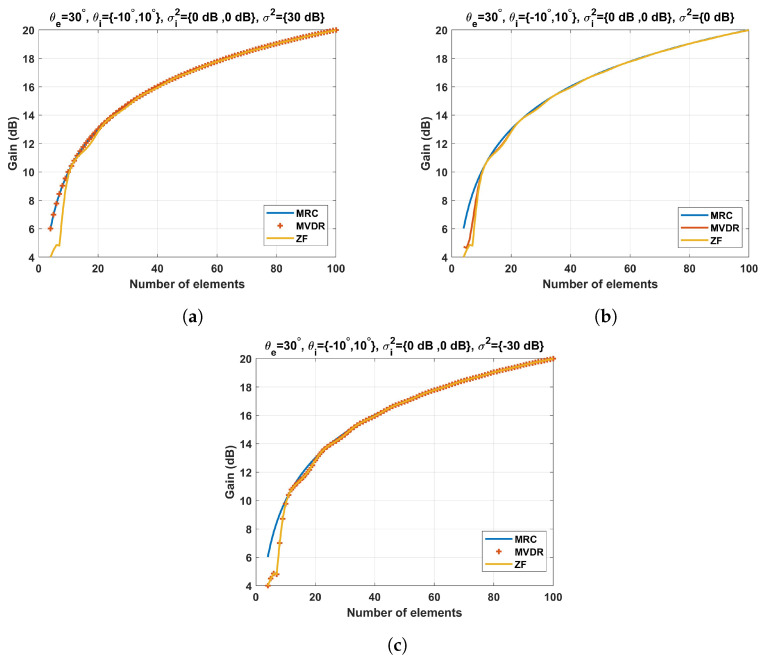
Beamforming techniques gain as a function of the number of array elements and the noise power: (**a**) $\sigma^{2}=30$ dB; (**b**) $\sigma^{2}=0$ dB; (**c**) $\sigma^{2}=-30$ dB.

Taking as a reference the desired steering angle, Figure 6 shows how the beamforming gain of the different techniques varies as a function of the number of array elements and the noise power. This gain is a scaled version of the numerator of both the SNR and the SINR; see (7) and (8), respectively. As anticipated in the analysis of Section 3.1, when the number of array elements *M* is large enough, the gain of the three strategies converges to *M*. However, when the number of array elements is scarce, slight discrepancies between the different techniques can be observed. As the MRC technique maximizes the desired signal power regardless of the interference signals, it presents a higher gain in the target direction.

Nevertheless, the interference signals are likely to remain as strong disturbances. On the contrary, the ZF does not present such high gain, but it completely removes the interference signals. On the other hand, the MVDR technique maximizes the ratio between the desired signal power and the undesired signal power (interference and noise). Therefore, the MVDR gain will depend on the noise level. When the noise level is small compared to the power of the desired signal, the MVDR gain approximates that of the ZF method (Figure 6c), whereas if the noise level is larger than the desired signal power, the MVDR gain tends to the MRC gain (Figure 6a). For intermediate noise values, the MVDR gain will be bounded by the MRC and the ZF gains until they converge to the number of array elements. It is worth noting that the ZF gain exhibits erratic behavior when the number of interference signals *k* is similar to the number of array elements *M*, as the spatial degrees of freedom available for beamforming are used to remove the undesired signals, sacrificing the array gain in favor of the stringent null constraints. This effect can be seen in Figure 6 (ZF gain line for a number of array elements smaller than 7).

### 4.3. Range–Doppler Map When Beamforming Is Applied before the CAF

In this subsection, the range–Doppler map corresponding to each beamforming strategy is evaluated when the CAF is processed after the beamforming. Accordingly, we evaluate if the different techniques remove or partially attenuate the echo signal of targets #1 and #3 while the strength of target #2 increases or, at least, remains the same. Moreover, we consider scenarios with different noise power levels.

**Figure 7:**
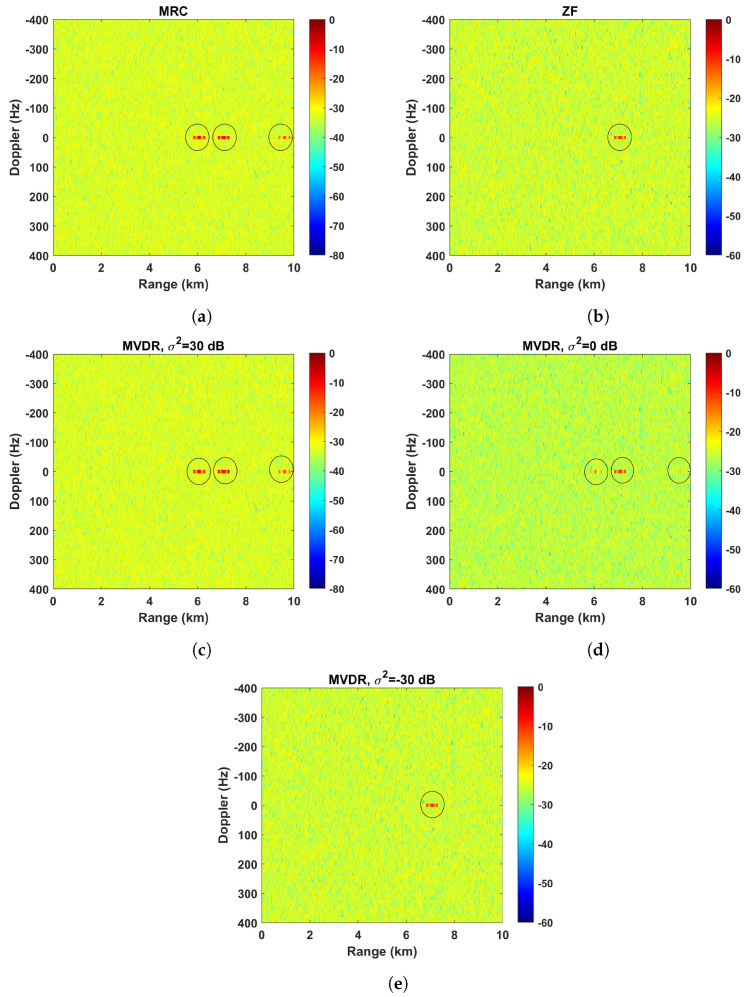
Range–Doppler map applying different beamforming techniques before the CAF: (**a**) MRC; (**b**) ZF; (**c**) MVDR ($\sigma^{2}=30$ dB); (**d**) MVDR ($\sigma^{2}=0$ dB); (**e**) MVDR ($\sigma^{2}=-30$ dB).

The range–Doppler maps corresponding to the MRC, MVDR, and ZF strategies are shown in Figure 7 for a fixed number of array elements M=4. As previously noted, the MRC beamforming strategy maximizes the desired signal power without considering the interference spatial signature, hence the range–Doppler map incorrectly shows the presence of the three targets (Figure 7a). On the opposite side, the ZF strategy completely removes the undesired target responses at the expense of reducing the gain in the steering direction (Figure 7b). In the case of the MVDR strategy, as it maximizes the ratio between the desired signal power and the interference and noise signal power, the dependence of the response with the noise power level appears. Thus, if the noise power is much larger than the interference signal power, the MVDR strategy exhibits the same behavior as the MRC strategy and, therefore, the three targets are visible in the range–Doppler map (Figure 7c).

Conversely, if the noise power is much smaller than the interference signal power, the MVDR strategy removes the undesired target responses just like the ZF strategy (Figure 7e). Finally, if the magnitude of the noise power is comparable to the magnitude of the interference signal power, the MVDR strategy attenuates the undesired target echoes, but without completely removing them with the aim of avoiding an excessive reduction on the gain in the desired signal direction (Figure 7d).

### 4.4. Range–Doppler Map When Beamforming Is Applied after the CAF

In this subsection, the range–Doppler map is evaluated when the respective beamforming strategies are applied after the CAF for each single array element. That is, *M* maps are processed and later combined to produce the actual range–Doppler map. When beamforming is applied after the CAF, it exploits the fact that signal contributions corresponding to the desired signal and to the interference present different signatures in the range–Doppler domain. As a consequence, they can be isolated and the spatial covariance matrices can be obtained for certain values of the range and the Doppler parameters. However, both the desired signal echo and the interference signal echoes can be extended over several ranges and/or Doppler bins, making the selection of interference and noise regions difficult unless accurate information regarding the scenario is used.

**Figure 8:**
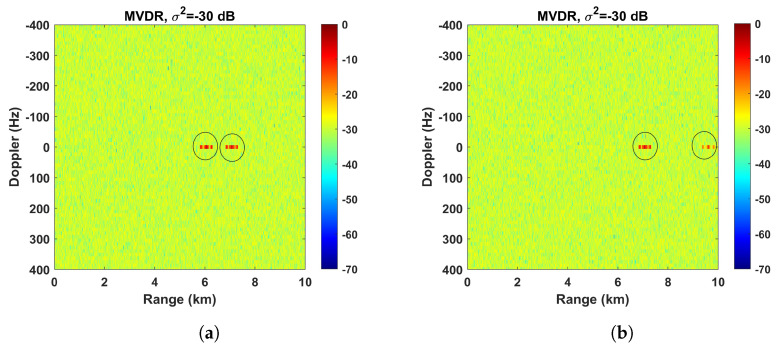
Range–Doppler map applying the MVDR beamforming technique after the CAF where the interference signals are not removed correctly: (**a**) target #1 is not removed; (**b**) target #3 is not removed.

A way to evaluate the performance of the strategy of applying the beamforming after the CAF is to study the acquisition of the interference and noise matrices R (Section 3.2), that is, how the covariance matrix is affected when the ratio between the interference signal power and the noise power level does not correspond to the true ratio. This might occur, for example, when the range–Doppler intervals are not chosen correctly. In this case, the coefficients estimated from scalar correlation functions such as $g_{d}\left(\tau ,l\right)$ are smaller than they should be, causing deviations in the SINR optimal structure for the covariance matrices. Even the relationship between the different undesired signals could be incorrect, leading to a similar result. Figure 8 shows the effects of incorrectly estimating these covariance matrices. For instance, when the covariance matrix belonging to the interference generated by target #1 is poorly estimated, this undesired target is not completely removed from the range–Doppler map (Figure 8a). The same effect can be observed when the signal strength belonging to the interference generated by target #3 is inaccurately estimated, as it is still present in the range–Doppler map (Figure 8b).

## 5. Open Issues and Future Research Directions

In this section, we pose some issues that hinder or limit the system’s performance, with the aim of providing a clear picture that helps to identify open problems and further improvements that might be addressed by the research community in the future.

### 5.1. Improving the Estimation Accuracy of the Covariance Matrices

It is clear from the analysis that obtaining precise information on the spatial features of the interference is critical to achieving good performance results. A small number of studies consider the use of the range–Doppler domain to obtain this information, with the sample covariance matrix being the most used strategy. We think that a possible line of research is the improvement of this estimation procedure, as the combination of range–Doppler–beam selection might help to characterize the spatial signatures of the targets and the interference. For instance, a multi-frequency joint sparse Bayesian learning method is proposed in [60], although it is only effective after removing interference and clutter components.

### 5.2. Exploiting Different IoOs to Enhance the Quality of the Reference Channel

It is clear that the utilization of an antenna array to sound the surveillance channel exhibits fundamental advantages. This rationale is also valid for the acquisition of the reference signal. As such, it might be useful to consider the utilization of these technologies to obtain the reference signal and how they can be exploited to improve detection. For example, two possible use cases are the employment of the same array to receive both the surveillance and reference channels, see, e.g., [29], or the coherent combination of the reference signals coming from different IoOs.

### 5.3. Simultaneous Detection of Multiple Targets

The detection of targets using a passive radar is an interesting problem, as can be seen from the amount of literature on this topic. However, most of the work focuses on scenarios where the detection capabilities of the system focus on searching for a single target. A more challenging scenario arises when multiple targets are present, especially when the number of targets is not available [63,64]. Smart employment of the scenario information and target attributes is the key to obtaining reasonable performance detection even without reference signals [65,66]. In this context, the use of antenna arrays is very helpful, as the angular discrimination of the different targets makes them easier to identify.

### 5.4. Combination with Temporal Processing Techniques

The successful use of temporal processing techniques such as ECA makes them an appealing resource that can be combined with both the angular and range–Doppler beamforming techniques. Accordingly, more specific versions of temporal processing techniques can be used, as they might be useful to remove some clutter or interference components if their range–Doppler–beam features make it possible to separate them from the signal of interest. Combining the two approaches might lead to solutions similar to that provided in STAP schemes, which is a minor trend in the context of passive radar [34,35,36].

### 5.5. Spherical-Wave Front for Passive Radar

When the antenna sizes and the target distances are comparable, the far-field assumption no longer applies, and the planar-wave front assumption is no longer valid [67]. This means that the traditional assumptions and methods employed to compute the beamformer and the achievable performances have to be revisited. This kind of scenario is meaningful, for instance, in indoor applications where the system implements a solution to locate objects or people [68,69]. Moreover, when moving to high frequencies such as mmWave radars [48], often equipped on cars that deploy a large number of small-sized antenna elements, the effects of this approach must be considered to efficiently use the radio-frequency spectrum.

### 5.6. Integration with Communication Systems

The efficient usage of the energy and the infrastructure employed in wireless communication systems is an actual trend in the research community. In particular, joint radar and communication systems have been regarded as a promising technology enabling communication with users and, at the same time, performing target location or tracking [70]. Nevertheless, the coexistence of two applications leads to the need for a balance between the performance obtained with both sub-systems. Therefore, obtaining the beamforming vectors is a challenging problem that must be carefully addressed, and it suffers from effects such as multi-user interference or target blockage. To alleviate these issues, and under the philosophy of reducing power consumption, the utilization of reconfigurable intelligent surface (RIS) is an appealing opportunity for these dual systems [71,72,73]. RISs are passive surfaces with a massive number of reconfigurable passive elements that allow a smart adaptation for the propagation environment [74]. Consequently, adopting this technology would provide additional capabilities in the context of passive radar, as it can be exploited as an additional and configurable IoO.

## 6. Conclusions

The use of passive radar in the context of non-cooperative target detection is an important current research issue. This article presents a review of the main beamforming procedures covered so far and develops these in the angular and range–Doppler domains in order to analyze their applicability and performance characteristics. The results obtained and supported by our numerical evaluations confirm that estimations and target detection are more manageable in the range–Doppler domain. However, this approach is computationally complex in contrast with the angular domain, which, in addition, is more flexible. This leads us to identify several open problems and further challenges to be addressed for the research community.

## Figures and Tables

**Table 1:** Summary of main beamforming strategies.

Name	Optimization Problem	Beamformer Expression
MRC	$\max|b^{H}{d|}^{2}$	b=ρd
MVDR	$\max\frac{|b^{H}{d|}^{2}}{b^{H}Rb}$	$b=\rho R^{-1}d$
ZF	$\max|b^{H}{d|}^{2}$ s.t. $b^{H}R=0$	$b=\rho (I_{M}-R{\left(R^{H}R\right)}^{-1}R^{H})d$

**Table 2:** Classification of main work on the reference list.

Domain		Beamforming Type			References
Angular	Range–Doppler	MRC	MVDR	ZF	
✓	✗	✓	✗	✗	[24,29,35,36,56]
✓	✗	✗	✓	✗	[24,30,33,37,57,58,59]
✓	✗	✗	✗	✓	[27,31,32,40]
✗	✓	✓	✗	✗	[60]
✗	✓	✗	✓	✗	[14,29,34,37,38,40,61,62]
✓	✓	✗	✓	✗	[35,36]

**Table 3:** Summary of main transmission parameters and bistatic resolutions.

Parameter	Value
Operating frequency	618 MHz
Acquisition time	85 ms
Signal bandwidth	12 MHz
Bistatic range resolution (maximum)	18 m
Bistatic Doppler resolution (maximum)	5.7 Hz
Elements distance (λ/2)	24 cm

## Data Availability

Not applicable.

## Abbreviations

The following abbreviations are used in this manuscript:

AoI	Area of Interest
AWGN	Additive White Gaussian Noise
CAF	Cross Ambiguity Function
CPI	Coherent Processing Interval
DAB	Digital Audio Broadcasting
DoA	Direction of Arrival
DPI	Direct-Path Interference
DVB-T	Digital Video Broadcasting–Terrestrial
DVB-S	Digital Video Broadcasting–Satellite
ECA	Extensive Cancellation Algorithm
ECM	Electronic Countermeasures
FM	Frequency Modulation
GNSS	Global Navigation Satellite System
GSM	Global System for Mobile Communications
IoO	Illuminator of Opportunity
LoS	Line-of-Sight
LTE	Long Term Evolution
MIMO	Multiple-Input Multiple-Output
MVDR	Minimum Variance Distortionless Response
mmWave	Millimeter Wave
MRC	Maximum Ratio Combining
MUSIC	Multiple Signal Classification
RIS	Reconfigurable Intelligent Surface
STAP	Space-Time Adaptive Processing
SINR	Signal-to-Interference-plus-Noise Ratio
SNR	Signal-to-Noise Ratio
ULA	Uniform Linear Array
ZF	Zero-Forcing
